# Establishment of Reference Intervals for Thyroid-Associated Hormones Using refineR Algorithm in Chinese Population at High-Altitude Areas

**DOI:** 10.3389/fendo.2022.816970

**Published:** 2022-02-11

**Authors:** Chaochao Ma, Jian Zhong, Yutong Zou, Zhijuan Liu, Honglei Li, Jinrong Pang, Xiaoxing Liu, Zejipuchi, Liping Tian, Li’an Hou, Danchen Wang, Xinqi Cheng, Ling Qiu

**Affiliations:** ^1^ Department of Laboratory Medicine, Peking Union Medical College Hospital, Peking Union Medical College & Chinese Academy of Medical Science, Beijing, China; ^2^ Department of Clinical Laboratory, People’s Hospital of Tibet Autonomous Region, Lhasa, China; ^3^ Department of Clinical Laboratory, Ali District People’s Hospital, Ali, China; ^4^ Department of Clinical Laboratory, Sang Zhu Zi District People’s Hospital, Shigatse, China; ^5^ Department of Clinical Laboratory, Maternal and Child Health Hospital, Nyingchi, China; ^6^ State Key Laboratory of Complex Severe and Rare Diseases, Peking Union Medical College Hospital, Chinese Academy of Medical Science and Peking Union Medical College, Beijing, China

**Keywords:** high altitude, reference interval, refineR algorithm, thyrotropin, thyroid hormones

## Abstract

**Objectives:**

Diagnosis of thyroid disease among individuals dwelling at high altitude remains a challenge. Reference intervals (RIs) for thyroid-associated hormones among Tibetans living at various high altitudes were established to improve diagnosis.

**Methods:**

One thousand two hundred eighty-one subjects were randomly recruited from Nyingchi, Shigatse/Lhasa, and Ali of Tibet. Thyroid-stimulating hormone (TSH), free triiodothyronine (FT3), and free thyroxine (FT4) were measured by the Cobas e601 electrochemiluminescence analyzer. We used multiple linear regression and variance component analysis to assess the effect of sex, age, and altitude on hormones. RIs were established by refineR algorithm and compared with those provided by the manufacturer.

**Results:**

Serum TSH was significantly lower in males than in females, while FT3 and FT4 were higher in males. Both FT3 and FT4 decreased with increasing age. FT3 increased with altitude, while TSH and FT4 were less influenced by altitude. The RI for TSH was 0.764–5.784 μIU/ml, while for FT4, the RIs were 12.36–19.38 pmol/L in females and 14.84–20.18 pmol/L in males. The RIs for FT3 at Nyingchi, Shigatse/Lhasa, and Ali in females were 4.09–4.98, 4.31–5.45, and 4.82–5.58 pmol/L, while in males, the values were 4.82–5.41, 4.88–5.95, and 5.26–6.06 pmol/L, respectively. The obtained RIs for TSH and FT4 were generally higher, while that for FT3 was narrower than the RIs provided by Cobas.

**Conclusions:**

Specific RIs were established for thyroid-associated hormones among Tibetans, which were significantly different from those provided by the manufacturer.

## Introduction

Thyroid disease is a prevalent health problem which may lead to potentially devastating healthy consequences (1). In China, the weighted prevalence of overt hyperthyroidism, overt hypothyroidism, subclinical hyperthyroidism, and subclinical hypothyroidism are known to be 0.78%, 1.02%, 0.44%, and 12.93%, respectively (2). Delayed diagnosis of thyroid disease can lead to adverse effects such as heart failure, atrial fibrillation, and mortality from other cardiovascular diseases, and redundant treatment due to misdiagnosis may also increase disease burden of patients or trigger adverse drug reactions (1, 3, 4). It was estimated that the rate of overdiagnosis of subclinical hypothyroidism was up to 16% due to physiological elevation of thyroid**-**stimulating hormone (TSH) (5, 6). Therefore, accurate and timely diagnosis is essential. The diagnosis of thyroid disease is predominantly based on laboratory measurements, of which the accuracy is mainly related to the applicability of reference interval (RIs). For some special groups such as the elderly (7), pregnant women (8), minority, and so on, general RIs may be not applicable. Similarity, habitants in the Tibet Plateau with an average altitude of more than 4,000 m may have their hypothalamic–pituitary–thyroid (HPT) axis altered for adaptation to severe cold, hypobaric hypoxia, sleep disorder, and other unfavorable conditions. This long-term adaptation may lead to thyroid-related hormone levels in Tibetans different from those in the plain (9–11). Even if the effects of sex, age (12–14), region, sampling time (15), and pregnancy status (16) on thyroid-related hormones are well discussed, however, only few articles (10, 11, 17) have assessed the influence of altitude on thyroid-associated hormones, let alone establishing altitude-specific RIs.

Currently, direct and indirect methods are the two sampling techniques applied to establish RIs. The direct method is the accepted standard method using *a priori* or posteriori sampling approach to provide a high internal validity and a minimal bias. However, the process is always costly and time-consuming and has poor feasibility (18). Since it is very difficult to define the apparently healthy individuals for specific groups such as the elderly, pregnant women, and people living in a special environment, the application of the direct method is restricted (18). Conversely, the indirect method with the simple and low-cost performance in a real-world environment utilizes the data mining technique to estimate the healthy distribution from the mixed distribution (19, 20). Thus, it can be used for the establishment of RIs when healthy individuals cannot be defined and obtained using direct sampling. The refineR algorithm has been previously proposed for the problem of “data mining” RIs (21). An open-source R code for the establishment of RIs using refineR algorithm has been developed by Ammer (21) and its effectiveness has been also proved. The core of the algorithm is still parameter combination and optimal search. Compared with the forward approach proposed by other indirect algorithms, the refineR algorithm adopts an inverse modeling approach to separate the healthy distribution of observed test results and identifies the optimal model for RI establishment. In short, using indirect methods such as refineR algorithm to establish RIs might be more preferable in Tibetan population, as the chronic exposure to high altitude may induce changes of thyroid-associated hormones for environmental adaptation (10, 11).

To help make more accurate clinical decisions on thyroid-related diseases, we used the variance component model to explore the effects of age, sex, and altitude on thyroid-associated hormones in Tibetan population. Furthermore, we established sex- and altitude-specific RIs for thyroid-related hormones in Tibetans based on the refineR algorithm and evaluated the application.

## Method and Material

### Subjects

From September 2016 to August 2018, we used a standard questionnaire to recruit participants at Ali (altitude I: 4,298–4,352 m), Shigatse/Lhasa (altitude II: 3,670–3,835 m), and Nyingchi (altitude III: about 2,900 m) of Tibet Autonomous Region in China. One thousand two hundred eighty-one indigenous Tibetan subjects were randomly enrolled in our study by the following criteria:

- Subjects self-report that they are currently in good health and have no major organ system disease,
- age ≥19 years,
- the subjects were Tibetan,
- subjects with no hospitalization in the past 6 months and no illness in the past 4 weeks, and
- subjects were required to have lived in Tibet for >1 year.

### Ethical Approval

This study has been approved by the Ethics Committee of the People’s Hospital of Tibet Autonomous Region (Approval No.ME-TBHP-2017-021) and the Ethics Committee of Peking Union Medical College Hospital (Approval No. S-K530). All the subjects had signed the informed consent form.

### Sample Collection

All subjects were requested to maintain on a normal diet and to avert night shifts or strenuous exercise 24 h before tests. After sitting for 10 to 15 min, fasting venous blood samples of subjects were drawn into red-capped procoagulant-containing Vacuette 5-mL tubes with gel (Greiner Bio-One, Kremsm€unster, Austria) by well-trained nurses and centrifuged at 3,000rpm for 10 min.

### Analytical Performance of Analytes

The levels of thyroid-related hormones including TSH, FT3, and FT4 were measured using the Cobas e601 electrochemiluminescence analyzer (Roche, Basel, Switzerland) with corresponding reagents, calibrators, and quality controls supplied by the manufacturer.

### Quality Control

In this study, sample quality was strictly controlled, and all sample collection, processing, and testing personnel were uniformly trained. Serum samples were centrifuged, packaged and frozen within 30 min after sampling, and transported to the Department of Laboratory Medicine of Peking Union Medical College Hospital for unified measurement through a cold chain transportation system with strict temperature control. For all test items, two levels of quality control were implemented before and after each batch test. Samples can be tested only after the quality control is qualified. In addition, the Department of Clinical Laboratory of Peking Union Medical College Hospital is accredited by both ISO15189 and CAP. All the above test items were evaluated by the National Health and Health Commission, and the results were all qualified.

### Data Cleaning and Statistical Analysis

Data cleaning and statistical analysis were performed using R programming language (V.4.0.5) and MedCalc Statistical software 18.116.6 (Mariakerke, Belgium). Shapiro–Wilk tests and frequency distribution histograms were used to describe the distributions of the items. If the variables satisfy a normal distribution, data were described as mean ± standard deviations and the Tukey method was used to identify outliers, whereas data rejecting the normality hypothesis were described as medians with quartiles. The Box–Cox method was applied to improve the normality of data before using the Tukey method to identify the outliers. Furthermore, when establishing reference intervals, the Box–Cox was performed again. The standardized regression coefficients of sex, altitude, and age were calculated by multiple linear regression. The variance component model was used to calculate the variation of thyroid-related hormones in gender, age, and altitude, that is, standard deviation or coefficient of variation. The residual standard deviation in the variance component model represents individual variation of thyroid-related hormones. Furthermore, standard deviation ratio (SDR) was expressed as (SDsex, SDaltitude, SDage)/SDresidual and was employed to judge whether the RI of thyroid-related hormones needs to be divided into several partitioning by sex, age, or altitude, and 0.4 is often used as a judgment threshold for thyroid-related hormones.

The refineR algorithm (21) was implemented using refineR package (version 1.0.0) for the aim of establishing RIs for thyroid-associated hormones. Two-sided *P <*0.05 was considered statistically significant.

## Results

### Baseline Information of Participants Enrolled in the Study

In total, 1,281 participants were enrolled in our study (Nyingchi: *n* = 363; Shigatse/Lhasa: *n* = 473; Ali: *n* = 445). Females accounted for 65.8%, 55.0%, and 51.9% of the recruits at altitudes III, II, and I, respectively. The median age of the Tibetan population at altitudes III, II, and I was 42, 42, and 34 years, respectively. The results of TPO-Ab and TG-Ab measured by Cobas are shown in **Table 1**.

**Table 1:** Baseline information of individuals enrolled in this study.

Index	Altitude III	Altitude II	Altitude I
*n*	363	473	445
Age (years)	42 (32, 51)	42 (32, 52)	34 (28, 43)
Sex (female%)	65.8%	55.0%	51.9%
TPO-Ab (IU/L)	13.25 (11.15, 16.21)	12.52 (10.02, 18.46)	14.51 (11.45, 9.28)
TG-Ab (IU/L)	10.00 (10.00, 10.54)	10.45 (10.00, 14.28)	14.00 (11.23, 18.97)

Altitude Ⅲ, Nyingchi (altitude: ~2,900 m); altitude Ⅱ, Shigatse/Lhasa (altitude: 3,670–3,835 m); altitude I, Ali (altitude: 4,298–4,352 m); TG-Ab, thyroglobulin antibody; TPO-Ab, thyroid peroxidase antibody.

### Effect of Sex, Altitude, and Age on Thyroid-Associated Hormones

**Figure 1:**
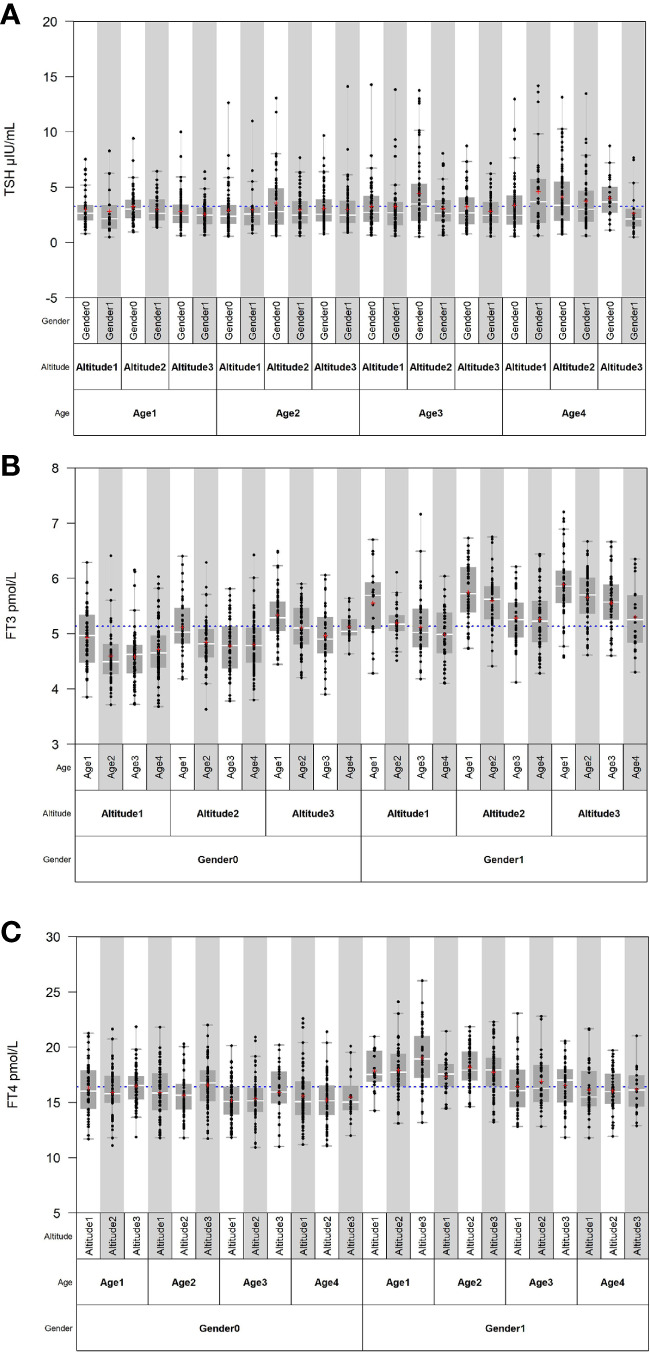
Distribution of thyroid-related hormones in Tibetan population by sex, age, and altitude. **(A–C)** Distribution of TSH, FT3, and FT4 in Tibetan population by sex, age, and altitude.

Source variations of each thyroid-related hormone were analyzed as shown in **Tables 2**, **3**. Sex has apparently effects on TSH, FT4, and FT3, showing that females have higher TSH levels but lower FT4 and FT3 levels than males. As shown in **Figure 1**, both FT3 and FT4 decreased with increasing age. Tibetans aged over 50 years have significant higher levels of TSH than those aged between 19 and 29 years (*P* < 0.001). FT3 increased with altitude (*P* < 0.001), while TSH and FT4 were less influenced by altitude. The results of multiple linear regression in **Table 2** and the variance component analysis in **Table 3** indicated that sex- and altitude-specific RIs for FT3 should be considered when the SDR ≥0.4. The RIs for FT4 should be partitioned by sex with SDRsex of 0.4.

**Table 2:** Effect of sex, altitude, and age on thyroid-associated hormones.

Index	Sex		Altitude				Age					
	*β*	*P*	Al 1		Al 2		A1		A2		A3	
			*β*	*P*	*B*	*P*	*β*	*P*	*β*	*P*	*B*	*P*
TSH	−0.060	0.038	0.077	0.026	−0.027	0.446	0.041	0.253	0.093	0.009	0.151	<0.001
FT3	0.425	<0.001	0.164	<0.001	0.309	<0.001	−0.185	<0.001	−0.257	<0.001	−0.250	<0.001
FT4	0.300	<0.001	0.000	0.991	0.076	0.020	−0.060	0.075	−0.216	<0.001	−0.235	<0.001

β, standardized regression coefficient; female is the reference for sex groups. Al 1 and Al 2 are the dummy variable of altitude, Nyingchi (altitude: ~2,900 m) is the reference level, Al 1 stands for Shigatse/Lhasa relative to Nyingchi and Al 2 stands for Ali relative to Nyingchi; A1, A2, and A3 are the dummy variable of age, 19~29 years is the reference level, A1 stands for 30~39 years relative to 19~29 years, A2 stands for 40~49 years relative to 19~29 years, A3 stands for age 50 or older relative to 19~29 years.

**Table 3:** Results of variance component model for thyroid-associated hormones.

Index	SDRresi	Sex		Altitude		Age	
		SD	SDR	SD	SDR	SD	SDR
TSH	2.18	0.33	0.2	0.00	0.0	0.34	0.2
FT3	0.50	0.38	0.8	0.21	0.4	0.17	0.3
FT4	2.14	0.96	0.4	0.23	0.1	0.71	0.3

### RIs of Thyroid-Associated Hormones for the Population at High Altitude

**Figure 2:**
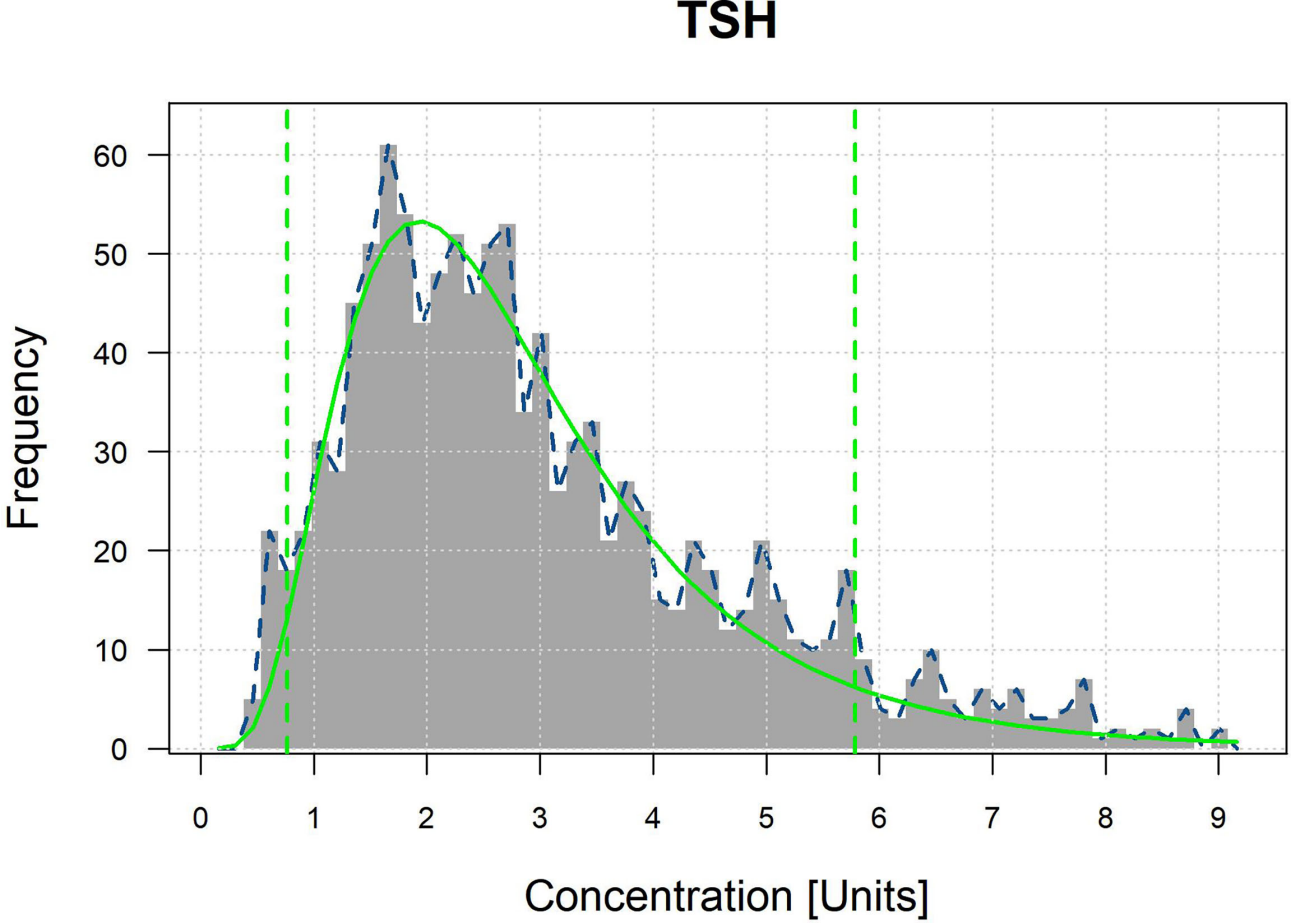
Optimal parametrical models calculated by refineR algorithm for TSH.

The optimal parametrical models for RI establishment calculated by the refineR algorithm are shown in **Figure 2** and **Appendix 1**. The specific results are shown in **Table 4**. RIs for FT3 were divided by sex and altitude, while RIs for FT4 were divided by sex. The RIs for FT3 and FT4 were overall higher in males than in females. RIs for FT3 elevated with increasing altitude as the RIs at altitudes III, II, and I were 4.09–4.98, 4.31–5.45, and 4.82–5.58 pmol/L in females and 4.82–5.41, 4.88–5.95, and 5.26–6.06 pmol/L in males. The total RIs for TSH, FT3, and FT4 were 0.764–5.784 μIU/ml, 4.01–6.23 pmol/L, and 12.19–20.70 pmol/L, respectively. Compared with the RIs provided by the manufacturer, the lower and upper limits of the obtained RIs for TSH and FT4 were higher, and the RI for FT3 was narrower.

**Table 4:** Reference interval of thyroid-associated hormones.

Index	Units	Group	refineR				Manufacturer	
			Lower limits of RI	90% CI	Upper limits of RI	90% CI	Lower limits of RI	Upper limits of RI
TSH	μIU/ml	Total	0.764	0.2276–0.9228	5.784	4.0818–8.2030	0.270	4.220
FT3	pmol/L	Total	4.01	3.909–4.240	6.23	5.701–6.554	3.10	6.80
		Female	3.98	3.803–4.298	5.79	5.370–6.077		
		Altitude III	4.09	3.777–4.424	4.98	4.769–5.324		
		Altitude II	4.31	4.051–4.628	5.45	5.119–5.762		
		Altitude I	4.82	4.411–5.048	5.58	5.379–5.848		
		Male	4.58	4.141–5.088	6.53	5.955–6.711		
		Altitude III	4.82	4.633–4.995	5.41	5.260–5.590		
		Altitude II	4.88	4.480–5.244	5.95	5.575–6.271		
		Altitude I	5.26	5.019–5.592	6.06	5.852–6.420		
FT4	pmol/L	Total	12.19	11.417–13.599	20.70	18.854–21.721	12.00	20.00
		Female	12.36	11.422–14.316	19.38	17.207–20.776		
		Male	14.84	12.407–16.318	20.18	18.890–21.825		

Altitude Ⅲ, Nyingchi (altitude: ~2,900 m); altitude Ⅱ, Shigatse/Lhasa (altitude: 3,670–3,835 m); altitude I, Ali (altitude: 4,298–4,352 m).

## Discussion

The current study utilized the refineR algorithm to formulate sex- and/or altitude-specific TSH, FT4 and FT3 RIs for indigenous Tibetans living at high altitude. Sex, age, and altitude had no effect on TSH levels in Tibetans and the RI for TSH was 0.764–5.784 μIU/ml. The level of FT3 was influenced by both sex and altitude, with the upper and lower limits of the RIs being higher for males than females and both increasing with altitude. The limits of TSH as well as FT4 were found to be significantly higher in our study as compared with those provided by the manufacturer. A narrower RI range for FT3 was also found. Therefore, to avoid misdiagnosis or underdiagnosis of thyroid disease, it is recommended to apply the appropriate specific RI for the Tibetan population.

Thyroid-related hormones are closely related to the metabolism of the body, short-term stress, or long-term adaptation. Due to the particularity of the living environment, Tibetan people may have hormone levels inconsistent with those in the plain areas. The present study found little effects of altitude on serum TSH among Tibetans, which is consistent with the results of an observation study (11). Relatively short half-life of TSH (17 to 93 min) (15), pulsatile secretion manners (15, 22) and feedback regulation of the HPT axis may roughly explain the phenomenon, although the exact mechanism is unclear. In euthyroid people, synthetic TSH is released in a manner combining a basal (non-pulsatile) and a pulsatile form and can be promptly cleared after action on the thyroid gland. The frequency, mass, and duration of a pulsatile can be rapidly adjusted to maintain the dynamic stability of TSH under the control of the HPT axis in response to environmental changes like hypobaric hypoxia, cold, and other harsh conditions on the plateau (10, 23). Meanwhile, the levels of thyroid-associated hormones were apparently elevated at higher altitude, with FT3 rising more obviously. Our results roughly concurred with previous studies which reported significant increases in thyroid-associated hormones for people following short-term or prolonged exposure to high-altitude environments (11, 17, 24, 25). Elevated thyroid-associated hormones in high-altitude environments facilitate the resistance of the body to the harsh environment of high altitude and seem to be independent on pituitary gland secretion of TSH (24, 26). Furthermore, altitude-dependent FT3 rise may also be relevant to physiological changes caused by the relative lack of iodine, as studies have shown that Tibetan regions have lower urinary iodine levels and a higher risk of iodine deficiency compared with mainland China (27, 28). Mouse models also showed that iodine deficiency could induce changes in deiodinase activity in the thyroid or peripheral tissues to affect the conversion of T4 to T3 (29), and make monocarboxylate transporter 8, one of the most important T3 relevant transporters, upregulated in the thyroid gland to transport more T3 into blood circulation (30).

Gender and age are important factors to be considered in the establishment of RIs for thyroid-related hormones. However, there is still a great controversy about whether the RIs should be partitioned by age (7, 12, 14, 31). In our study, the impact of age on RIs for TSH, FT3, and FT4 in Tibetans was modest. Although it was clear from the results that TSH levels were significant higher in the group aged over 50 years than in the group aged between 19 and 29 years, the RI could not be partitioned by age in our study and the RI for TSH was 0.764–5.784 μIU/ml. Zhai et al., using the same instrument as we did, reported that the RIs for TSH in Chinese plain population <65 and ≥65 years were 0.76–6.57 and 0.75–8.86 mIU/L, respectively (14). Difference in the upper limits may be explained by the age composition of the population living at different altitudes in China, as in the case of the three regions of Tibet, where the proportion of people aged >65 years was only approximately 5% (32). Obvious discrepancies were observed for TSH, FT3, and FT4 levels between males and females, although the effect of sex on TSH level was not as remarkable as those on FT3 or FT4, which was consistent with previous studies (14, 27, 33, 34). Thus, sex-specific RIs for FT4 and FT3, but not for TSH, were proposed in our study and results showed that both the upper and lower limits of RIs for FT3 and FT4 were higher in males than in females, which can be interpreted by the potential effect of sex hormones on the HPT axis (10, 35).

At the end of this study, we compared the RIs calculated by the refineR algorithm with those proposed by the manufacturer. Higher upper and lower reference limits were found in Tibetans for TSH and FT4 (TSH: 0.764–5.784 *vs.* 0.270–4.220 μIU/ml, FT4: 12.19–20.70 *vs.* 12.00–20.00 pmol/L), as well as a narrower range for FT3 (4.01–6.23 *vs.* 3.10–6.80 pmol/L). These discrepancies suggested that the direct use of the RIs of the manufacturer may lead to underdiagnosis of hyperthyroidism or overdiagnosis of subclinical hypothyroidism. The study highlights the importance of establishing appropriated RIs in high-altitude laboratories by an indirect method like the refineR algorithm. However, it is important that 90% CI of RIs show that the widths of CI of FT3 and FT4 are suitable. However, the 90% CI of the upper limit of reference interval for TSH is relatively wide, possibly due to the large variation of the TSH for populations at high altitude.

Our research has both advantages and disadvantages. Firstly, it is the first multicenter cross-sectional study to set up RIs for thyroid-related hormones in Tibetan population dwelling at the plateau, and the refineR algorithm was creatively used under this situation. Secondly, a detailed questionnaire was used to assist in screening subjects from Nyingchi, Shigatse, Lhasa, and Ali. Unequivocally, there are limitations in the current study, such as the lack of thyroid ultrasonography results and the utilization of only a single testing system. However, the refineR algorithm can help identify healthy individuals from a distribution mixed with a small proportion of pathological individuals and then obtain RIs according to the optimal parameter models. Considering that conventional definitions of apparent health, such as normal blood pressure and BMIs in the normal range, may not be applicable to high-altitude dwellers, the method in our study may be a preference to establish RIs. In addition, the RIs established for thyroid-related hormones may be only applicable to the Roche platform, as non-negligible discrepancy among assay methodologies has been revealed (13). Overall, we have currently established RIs for Tibetans living at high altitude for generations, and further studies should use real-world data to compare the prevalence of thyroid disease in populations dwelling at different altitudes for generations.

## Conclusion

Altitude- and/or sex-specific RIs for thyroid-related hormones were established in Chinese people living at high-altitude areas based on the refineR algorithm. Significant differences have been found while comparing the obtained RIs with the RIs provided by the manufacturer. Therefore, establishment of specific RIs according to regional characteristics of Tibet should be recommended to avoid underdiagnosis or misdiagnosis of thyroid disease.

## Data Availability Statement

The original contributions presented in the study are included in the article/**Supplementary Material** Further inquiries can be directed to the corresponding author.

## Ethics Statement

The studies involving human participants were reviewed and approved by the Ethics Committee of the People’s Hospital of Tibet Autonomous Region (Approval No.ME-TBHP-2017-021) and the Ethics Committee of Peking Union Medical College Hospital (Approval No. S-K530). The patients/participants provided their written informed consent to participate in this study.

## Author Contributions

LQ designed the study. CM analyzed the data. CM, JZ, and YZ wrote this manuscript. LH, DW, and CM performed detection of the thyroid-related hormones. XC, LQ, and DW made suggestions for the revision of the manuscript. HL, JP, ZL, XL, ZP, and LT helped in participant recruitment and sample collection. All authors reviewed the manuscript. All authors contributed to the article and approved the submitted version.

## Funding

The work was supported by the Special Foundation Project for Human Resources Development of the Tibet Autonomous Region (2016000), Science and Technology Program of the Tibet Autonomous Region (2015XZ01G20), Beijing Key Clinical Specialty for Laboratory Medicine - Excellent Project (No. ZK201000) and Capital’s Funds for Health Improvement and Research (CFH-2020-1-4014).

## Conflict of Interest

The authors declare that the research was conducted in the absence of any commercial or financial relationships that could be construed as a potential conflict of interest.

## Publisher’s Note

All claims expressed in this article are solely those of the authors and do not necessarily represent those of their affiliated organizations, or those of the publisher, the editors and the reviewers. Any product that may be evaluated in this article, or claim that may be made by its manufacturer, is not guaranteed or endorsed by the publisher.
