# Supplementary material for: Establishment of Reference Intervals for Thyroid-Associated Hormones Using refineR Algorithm in Chinese Population at High-Altitude Areas
Source: Front Endocrinol (Lausanne). 2022 Feb 11;13:816970. doi: 10.3389/fendo.2022.816970 (PMC8874314; doi:10.3389/fendo.2022.816970)
Supplement: Supplementary file 1 [file DataSheet_1.docx]

***Supplementary Material***

## Supplementary Figures

**Supplementary Figure 1.** Appendix 1 Optimal parametrical models calculated by refine R algorithm for FT4 and FT3 in different sex groups.


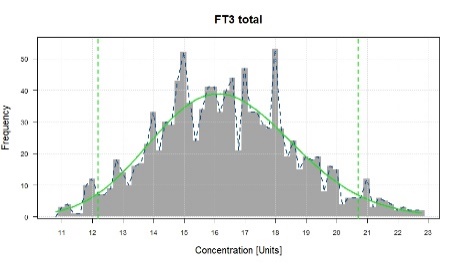
**A**
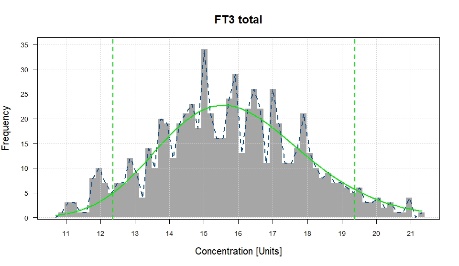

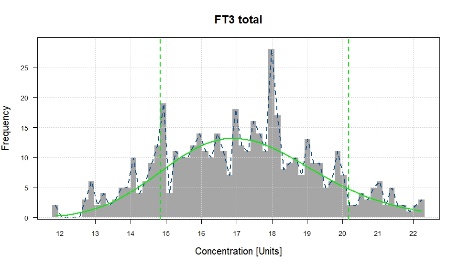


Total Female Male

**
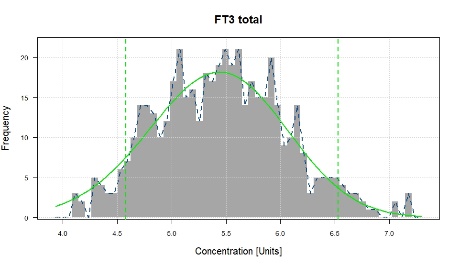

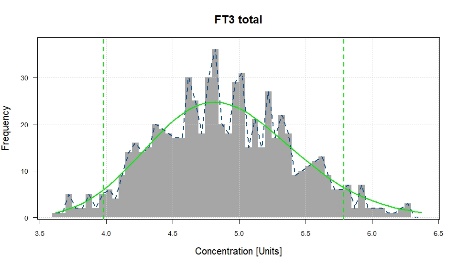
**
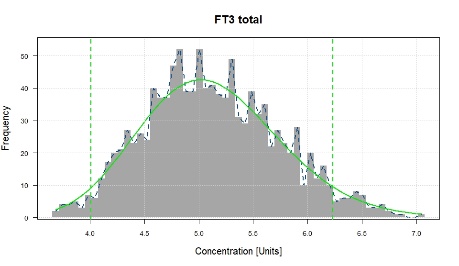
**B**

Total Female Male

A stands for the optimal parameter models corresponding to the FT4 RIs established for total, female and male Tibetan; B stands for the optimal parameter models corresponding to the FT3 RIs established for total, female and male Tibetan;

**Supplementary Figure 2.** Optimal parametrical models calculated by refine R algorithm for FT3 in different altitude groups.

A


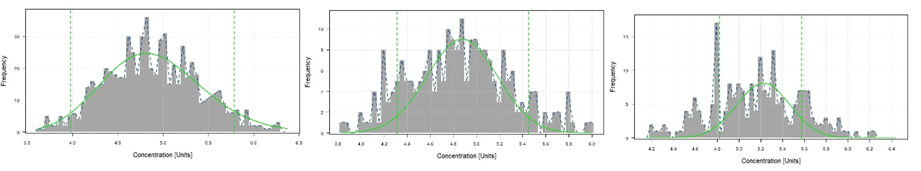
AltitudeⅢ AltitudeⅡ Altitude I


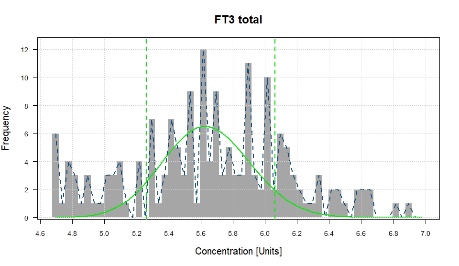
B
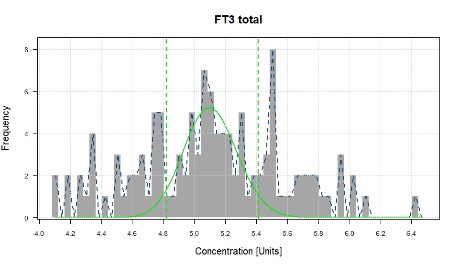

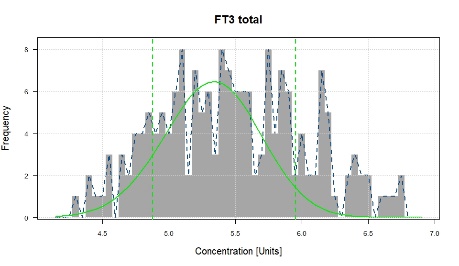


AltitudeⅢ AltitudeⅡ Altitude I

A and B represent, from left to right, the optimal parameter models for female and male Tibetan population when establishing FT3 RIs at Altitude III, II and I. AltitudeⅢ, Nyingchi (altitude: ~2900 m); AltitudeⅡ, Shigatse/Lhasa (altitude: 3670-3835 m); Altitude I, Ali (altitude: 4298-4352 m).
